# The Ageing Brain in India: Understanding the Social Determinants of Cognition

**DOI:** 10.1002/alz70857_104399

**Published:** 2025-12-24

**Authors:** Gauri Mullerpattan, Vidhya R, Abhishek Mensegere Lingegodwa, Prathima Arvind, Albert Stezin, Jonas S Sundarakumar, Thomas Gregor Issac

**Affiliations:** ^1^ Centre for Brain Research, Indian Institute of Science, Bangalore, Karnataka, India; ^2^ CBR, Bangalore, Karnataka, India

## Abstract

**Background:**

Cognitive ageing research has largely focused on high‐income countries, with limited representation from South Asia, particularly India, despite its rapidly ageing population. Given the distinct sociocultural and economic factors influencing ageing in this region, understanding how age, gender, education, occupation, and income impact cognitive performance is crucial for developing contextually relevant interventions. This study examines these determinants in shaping cognitive function among healthy older adults in India.

**Method:**

A cross‐sectional sample of 1,626 healthy adults aged 45 years and older from the Tata Longitudinal Study of Ageing (TLSA) cohort was analysed. Socioeconomic status (SES) was classified using the Kuppuswamy Socioeconomic Scale (Revised), incorporating education, occupation, and income. Cognition was assessed using the ACE III and COGNITO Neuropsychological Battery. Mann‐Whitney U tests examined cognitive differences based on age, gender, and SES.

**Result:**

Significant age‐related cognitive decline was observed, with older participants performing worse in global cognition (*p* < 0.001), memory (*p* < 0.001), fluency (*p* = 0.013), visuospatial skills (*p* = 0.001), and executive function (*p* < 0.001). Reaction time also slowed with increasing age (*p* < 0.001). Gender differences showed that males outperformed females in visuospatial tasks (*p* < 0.001), attention (*p* < 0.001), and executive function (*p* < 0.001). In contrast, females excelled in memory recall (*p* < 0.001), verbal fluency (*p* < 0.001), and categorical fluency (*p* < 0.001). Reaction time was significantly slower in females (*p* < 0.001). Economic determinants played a crucial role. Individuals from higher SES backgrounds performed significantly better across cognitive domains, including global cognition (*p* < 0.001), memory (*p* < 0.001), fluency (*p* < 0.001), and processing speed (*p* < 0.001). Higher educational attainment and professional occupations were associated with superior executive function and working memory. Middle‐class individuals exhibited slower reaction times (*p* < 0.001) and lower episodic memory recall (*p* < 0.001).

**Conclusion:**

The findings highlight the relationship between socioeconomic disparities and cognitive functioning. Higher education, professional occupations, and income were linked to better cognitive outcomes, underscoring the need for region‐specific cognitive health interventions. Addressing socioeconomic disparities in ageing is essential for globalising public health policies and promoting healthy cognitive ageing.

